# Brain morphometry in former American football players: findings from the DIAGNOSE CTE research project

**DOI:** 10.1093/brain/awae098

**Published:** 2024-03-27

**Authors:** Hector Arciniega, Zachary H Baucom, Fatima Tuz-Zahra, Yorghos Tripodis, Omar John, Holly Carrington, Nicholas Kim, Evdokiya E Knyazhanskaya, Leonard B Jung, Katherine Breedlove, Tim L T Wiegand, Daniel H Daneshvar, R Jarrett Rushmore, Tashrif Billah, Ofer Pasternak, Michael J Coleman, Charles H Adler, Charles Bernick, Laura J Balcer, Michael L Alosco, Inga K Koerte, Alexander P Lin, Jeffrey L Cummings, Eric M Reiman, Robert A Stern, Martha E Shenton, Sylvain Bouix

**Affiliations:** Psychiatry Neuroimaging Laboratory, Brigham and Women’s Hospital, Harvard Medical School, Boston, MA 02145, USA; Department of Rehabilitation Medicine, NYU Grossman School of Medicine, New York, NY 10016, USA; NYU Concussion Center, NYU Langone Health, New York, NY 10016, USA; Department of Biostatistics, Boston University School of Public Health, Boston, MA 02118, USA; Department of Biostatistics, Boston University School of Public Health, Boston, MA 02118, USA; Department of Biostatistics, Boston University School of Public Health, Boston, MA 02118, USA; Psychiatry Neuroimaging Laboratory, Brigham and Women’s Hospital, Harvard Medical School, Boston, MA 02145, USA; Department of Rehabilitation Medicine, NYU Grossman School of Medicine, New York, NY 10016, USA; NYU Concussion Center, NYU Langone Health, New York, NY 10016, USA; Psychiatry Neuroimaging Laboratory, Brigham and Women’s Hospital, Harvard Medical School, Boston, MA 02145, USA; Psychiatry Neuroimaging Laboratory, Brigham and Women’s Hospital, Harvard Medical School, Boston, MA 02145, USA; Psychiatry Neuroimaging Laboratory, Brigham and Women’s Hospital, Harvard Medical School, Boston, MA 02145, USA; Psychiatry Neuroimaging Laboratory, Brigham and Women’s Hospital, Harvard Medical School, Boston, MA 02145, USA; cBRAIN, Department of Child and Adolescent Psychiatry Psychosomatics and Psychotherapy, University Hospital Ludwig-Maximilians-Universität, Munich, Bavaria 80336, Germany; Center for Clinical Spectroscopy, Department of Radiology, Brigham and Women’s Hospital, Harvard Medical School, Boston, MA 02115, USA; Psychiatry Neuroimaging Laboratory, Brigham and Women’s Hospital, Harvard Medical School, Boston, MA 02145, USA; cBRAIN, Department of Child and Adolescent Psychiatry Psychosomatics and Psychotherapy, University Hospital Ludwig-Maximilians-Universität, Munich, Bavaria 80336, Germany; Department of Physical Medicine and Rehabilitation, Harvard Medical School, Boston, MA 02115, USA; Department of Physical Medicine and Rehabilitation, Massachusetts General Hospital, Boston, MA 02114, USA; Department of Physical Medicine and Rehabilitation, Spaulding Rehabilitation Hospital, Boston, MA 02129, USA; Psychiatry Neuroimaging Laboratory, Brigham and Women’s Hospital, Harvard Medical School, Boston, MA 02145, USA; Department of Anatomy and Neurobiology, Boston University Chobanian & Avedisian School of Medicine, Boston, MA 02118, USA; Psychiatry Neuroimaging Laboratory, Brigham and Women’s Hospital, Harvard Medical School, Boston, MA 02145, USA; Psychiatry Neuroimaging Laboratory, Brigham and Women’s Hospital, Harvard Medical School, Boston, MA 02145, USA; Department of Radiology, Brigham and Women’s Hospital, Harvard Medical School, Boston, MA 02115, USA; Department of Psychiatry, Massachusetts General Hospital, Boston, MA 02114, USA; Psychiatry Neuroimaging Laboratory, Brigham and Women’s Hospital, Harvard Medical School, Boston, MA 02145, USA; Department of Neurology, Mayo Clinic College of Medicine, Mayo Clinic Arizona, Scottsdale, AZ 85259, USA; Cleveland Clinic Lou Ruvo Center for Brain Health, Las Vegas, NV 89106, USA; Department of Neurology, University of Washington, Seattle, WA 98195, USA; Department of Neurology, NYU Grossman School of Medicine, New York, NY 10017, USA; Department of Population Health, NYU Grossman School of Medicine, New York, NY 10017, USA; Department of Ophthalmology, NYU Grossman School of Medicine, New York, NY 10017, USA; Department of Neurology, Boston University Alzheimer’s Disease Research Center and CTE Center, Boston University Chobanian & Avedisian School of Medicine, Boston, MA 02118, USA; Psychiatry Neuroimaging Laboratory, Brigham and Women’s Hospital, Harvard Medical School, Boston, MA 02145, USA; cBRAIN, Department of Child and Adolescent Psychiatry Psychosomatics and Psychotherapy, University Hospital Ludwig-Maximilians-Universität, Munich, Bavaria 80336, Germany; Department of Psychiatry, Massachusetts General Hospital, Boston, MA 02114, USA; Graduate School of Systemic Neurosciences, Ludwig-Maximilians-Universität, 82152 Munich, Bavaria, Germany; Center for Clinical Spectroscopy, Department of Radiology, Brigham and Women’s Hospital, Harvard Medical School, Boston, MA 02115, USA; Department of Radiology, Brigham and Women’s Hospital, Harvard Medical School, Boston, MA 02115, USA; Chambers-Grundy Center for Transformative Neuroscience, Pam Quirk Brain Health and Biomarker Laboratory, Department of Brain Health School of Integrated Health Sciences, University of Nevada Las Vegas, Las Vegas, NV 89154, USA; Banner Alzheimer’s Institute and Arizona Alzheimer’s Consortium, Phoenix, AZ 85006, USA; Department of Psychiatry, University of Arizona, Phoenix, AZ 85004, USA; Department of Psychiatry, Arizona State University, Phoenix, AZ 85008, USA; Neurogenomics Division, Translational Genomics Research Institute and Alzheimer’s Consortium, Phoenix, AZ 85004, USA; Department of Anatomy and Neurobiology, Boston University Chobanian & Avedisian School of Medicine, Boston, MA 02118, USA; Department of Neurology, Boston University Alzheimer’s Disease Research Center and CTE Center, Boston University Chobanian & Avedisian School of Medicine, Boston, MA 02118, USA; Department of Neurosurgery, Boston University Chobanian & Avedisian School of Medicine, Boston, MA 02118, USA; Psychiatry Neuroimaging Laboratory, Brigham and Women’s Hospital, Harvard Medical School, Boston, MA 02145, USA; Department of Radiology, Brigham and Women’s Hospital, Harvard Medical School, Boston, MA 02115, USA; Department of Psychiatry, Massachusetts General Hospital, Boston, MA 02114, USA; Department of Software Engineering and Information Technology, École de technologie supérieure, Université du Québec, Montréal, QC H3C 1K3, Canada

**Keywords:** neuroimaging, structural MRI, sports-related head injury, repetitive head impact, former American football players

## Abstract

Exposure to repetitive head impacts in contact sports is associated with neurodegenerative disorders including chronic traumatic encephalopathy (CTE), which currently can be diagnosed only at post-mortem. American football players are at higher risk of developing CTE given their exposure to repetitive head impacts. One promising approach for diagnosing CTE *in vivo* is to explore known neuropathological abnormalities at post-mortem in living individuals using structural MRI.

MRI brain morphometry was evaluated in 170 male former American football players ages 45–74 years (*n* = 114 professional; *n* = 56 college) and 54 same-age unexposed asymptomatic male controls (*n* = 54, age range 45–74). Cortical thickness and volume of regions of interest were selected based on established CTE pathology findings and were assessed using FreeSurfer. Group differences and interactions with age and exposure factors were evaluated using a generalized least squares model. A separate logistic regression and independent multinomial model were performed to predict each traumatic encephalopathy syndrome (TES) diagnosis, core clinical features and provisional level of certainty for CTE pathology using brain regions of interest.

Former college and professional American football players (combined) showed significant cortical thickness and/or volume reductions compared to unexposed asymptomatic controls in the hippocampus, amygdala, entorhinal cortex, parahippocampal gyrus, insula, temporal pole and superior frontal gyrus. *Post hoc* analyses identified group-level differences between former professional players and unexposed asymptomatic controls in the hippocampus, amygdala, entorhinal cortex, parahippocampal gyrus, insula and superior frontal gyrus. Former college players showed significant volume reductions in the hippocampus, amygdala and superior frontal gyrus compared to the unexposed asymptomatic controls. We did not observe Age × Group interactions for brain morphometric measures. Interactions between morphometry and exposure measures were limited to a single significant positive association between the age of first exposure to organized tackle football and right insular volume. We found no significant relationship between brain morphometric measures and the TES diagnosis core clinical features and provisional level of certainty for CTE pathology outcomes.

These findings suggested that MRI morphometrics detect abnormalities in individuals with a history of repetitive head impact exposure that resemble the anatomic distribution of pathological findings from post-mortem CTE studies. The lack of findings associating MRI measures with exposure metrics (except for one significant relationship) or TES diagnosis and core clinical features suggested that brain morphometry must be complemented by other types of measures to characterize individuals with repetitive head impacts.

## Teaser

Exposure to repetitive head impacts is associated with an increased risk of chronic traumatic encephalopathy (CTE). Using structural MRI, Arciniega *et al.* reveal reduced cortical thickness and/or volume in living former American football players compared to unexposed asymptomatic controls in regions associated with CTE at post-mortem.

## Introduction

Chronic traumatic encephalopathy (CTE) is a neurodegenerative disease associated with a history of repetitive head impact (RHI) exposure characterized by perivascular hyperphosphorylated tau (p-tau) depositions in neurons with or without astrocytes at the depth of the cerebral sulci.^1-3^ In the initial stages of CTE, p-tau depositions are primarily observed in frontotemporal brain regions and later progress to medial temporal lobes followed by widespread distribution across the brain.^4-7^ The p-tau depositions within these regions have been linked to cognitive deficits behaviour changes mood deficits and in a small number of cases motor deficits.^8^

CTE pathology has been found at post-mortem in the brains of contact sports athletes such as American football players who are exposed to RHIs.^9-14^ However, there are no currently available *in vivo* diagnostic markers of CTE, meaning a diagnosis can be made only after death. There is thus a need to establish *in vivo* diagnostic biomarkers for CTE so that interventions can be developed to slow progression or prevent the disease.

Neuropathological studies of athletes involved in contact sports have led to the McKee CTE Staging Scheme defined by four pathological stages of CTE, stages 1 (mild) to 4 (severe).^7,15^ In stage 1, the pathology is localized to the superior dorsolateral and inferior frontal cortices. Here, the deposition of p-tau is largely found in the sulci of brain regions located around blood vessels.^1,2,4-6,16^ In stage 2, other macroscopic changes are observed, including mild enlargement of the frontal horns of the lateral ventricles and the third ventricles, and in some cases the presence of a cavum septum pellucidum. In stage 3, there is a reduction in brain weight, mild frontal and temporal atrophy, and further enlargement of the lateral and third ventricles. Importantly, in stage 3, neurofibrillary tangles are visible in the olfactory bulb, hippocampus, entorhinal cortex, amygdala, hypothalamus and mammillary bodies. Stage 4 is characterized by more widespread regional brain pathology and includes decreases in myelinated nerve fibres and axonal dystrophy.^1,2,4-6^ Overall, the pathology of CTE is well categorized at post-mortem, which allows us to target regions for *in vivo* neuroimaging analyses and possible *in vivo* diagnoses that correspond to those identified with post-mortem studies.

The clinical features associated with neuropathologically diagnosed CTE are characterized through the 2021 National Institute of Neurological Disorders and Stroke (NINDS) consensus diagnostic criteria for traumatic encephalopathy syndrome (TES).^17^ A diagnosis of TES requires a substantial RHI exposure, core clinical features of cognitive impairment (in episodic memory and/or executive functioning) and/or neurobehavioural dysregulation, a progressive course and that the core clinical features are not fully accounted for by other neurological, psychiatric or medical conditions.^17^ Importantly, the consensus panelists agreed that *in vivo* biomarker development for CTE was not sufficiently mature to be included in the diagnostic criteria. Possible biomarkers can include the use of PET imaging with specialized radiotracers that bind to CTE tau isoforms, CSF or blood analytes for p-tau markers, functional connectivity and neurochemical metabolism.^17-19^ Here we focus on structural anatomical neuroimaging biomarkers that have the potential to establish underlying biological links between RHI TES and CTE neuropathology.

Specific demographic and RHI exposure variables that may lead to CTE remain largely unknown. Exposure to RHIs is key to the development of CTE, although not everyone who is exposed to RHIs will develop the disease.^2,9^ Understanding how demographic (e.g. age)^20^ and exposure metrics (e.g. total years in football, age of first exposure, cumulative head impact index)^21-23^ are associated with RHI, and the development of CTE is therefore important for the diagnosis of CTE during the lifetime, understanding disease progression and developing strategies for treatment and prevention.

One promising approach to establish *in vivo* biomarkers of CTE is to use structural MRI to detect changes that may reflect those observed in post-mortem studies. Tauopathy findings at post-mortem suggest that frontal and temporal lobe brain regions are most likely impacted and can be explored *in vivo* in structural neuroimaging studies.^1,2,4-6,15^ In addition, a recent study of antemortem structural MRI in confirmed cases of CTE found atrophy to be most severe in the frontal anterior temporal and medial temporal lobes compared to controls.^24^ These findings led us to hypothesize that brain regions known to be impacted by tauopathy in CTE may display thickness and/or volume reductions observable in structural *in vivo* MRI.^24^ Accordingly, we predicted that cortical thickness and/or volume may be sensitive measures that will allow us to detect subtle group-level changes that may be consistent with CTE pathology at post-mortem.

In this study, we made three main contributions. First, we characterized *in vivo* cortical and subcortical morphometric changes in former American football players in regions known to be associated with post-mortem CTE pathology. Here, as described earlier, we focused on regions that can be segmented from MRI and that are hallmarks of CTE pathology, including the superior frontal gyrus, caudal middle frontal gyrus, rostral middle frontal gyrus, entorhinal cortex, parahippocampal gyrus, insula, temporal pole, amygdala, hippocampus and hypothalamus. First, we tested group-level differences between former American football players and healthy unexposed asymptomatic controls and further dichotomized the former American football player sample into two groups (former college players and former professional players). Second, we analysed the association between brain morphometry and age as well as exposure measures that may be associated with the development of CTE (age of first exposure to football, total years in football and cumulative head impact index measures, including frequency, linear acceleration and rotational force). Third, we studied the link between the identified abnormalities and TES diagnosis, TES core clinical features of cognitive impairment and neurobehavioural dysregulation and the provisional levels of certainty for CTE pathology.

## Materials and methods

### Study design and participants

This study is part of the Diagnostics Imaging And Genetics Network for the Objective Study and Evaluation of Chronic Traumatic Encephalopathy (DIAGNOSE CTE) Research Project. DIAGNOSE CTE is a large multi-site study where the protocol includes neurological and psychiatric examinations, assessment of exposure to RHI, neuropsychological testing, self- and informant-report measures of neuropsychiatric symptoms, lumbar puncture and blood draw (for fluid biomarkers), and neuroimaging (PET, structural diffusion and functional MRI and magnetic resonance spectroscopy) in former professional players, former college football players and healthy unexposed asymptomatic controls.^25^ The study and its procedures were approved by the Boston University Medical Campus, Mayo Clinic, Banner Alzheimer’s Institute, New York University (NYU) Medical Center-Langone and Brigham and Women’s Hospital Institutional Review Boards. All participants provided written informed consent before enrolment. All baseline data were collected before the coronavirus-2019 pandemic.

Overall, there are 240 participants in DIAGNOSE CTE, including 180 former American football players (120 former professional players and 60 former college players) and 60 same-age men without a history of contact sports, RHI exposure or TBI and who denied cognitive or psychiatric symptoms at telephone screening. Data from 16 participants were excluded from the current analyses because of poor-quality or incomplete structural MRI. Data from four unexposed asymptomatic control participants were removed as in follow-up interviews they reported having a history of pre-existing psychiatric conditions and treatment before the baseline enrolment period or participated in high school football. The final sample consisted of 170 former American football players (114 former professional and 56 former college players) and 54 unexposed controls, resulting in a total of 224 participants; see Table 1 for detailed demographics.

**Table 1:** Cohort characteristics

	Former football players (*n* = 170)	Former professional players (*n* = 114)	Former college players (*n* = 56)	Unexposed asymptomatic controls (*n* = 54)
Primary demographics				
Age, years	57.2 (8.1) [45–74]	59.2 (7.8) [45–74]	53.2 (7.4) [45–74]	59.4 (8.5) [45–74]
BMI, kg/m^2^	32.7 (4.7) [22.8–47.4]	32.1 (4.5) [22.8–47.4]	33.9 (4.9) [23.6–44.6]	31 (4.6) [23.7–43.5]
Education, years	16.7 (1.5) [15–27]	16.6 (1.1) [15–21]	17.1 (2) [15–27]	17.2 (3.4) [13–30]
Apolipoprotein 4 carriers^a^	48 (28.2%)	30 (26.3%)	18 (32.1%)	10 (18.5%)
Race				
White	108 (63.5%)	64 (56.14%)	44 (78.6%)	34 (63%)
Black/African American	59 (34.7%)	48 (42.11%)	11(19.6%)	19 (35.2%)
American Indian/Alaska Native	0 (0%)	0 (0%)	0 (0%)	0 (0%)
Asian	0 (0%)	0 (0%)	0 (0%)	0 (0%)
Native Hawaiian/Other Pacific Islander	0 (0%)	0 (0%)	0 (0%)	1 (1.8%)
Multiple races	3 (1.8%)	2 (1.75%)	1 (1.8%)	0 (0%)
Exposure to RHIs				
Time in football, years	16 (4.3) [6–25]	18 (3.4) [4–23]	11.6 (2.6) [6–17]	–
Age of first exposure, years	11.1 (2.8) [4–18]	14.8 (4.1) [4–18]	12.2 (3.4) [5–16]	–
Cumulative head impact index seasons				
Frequency	10 869 (4689) [3560–28 020]	12 014 (5055) [3560–28 020]	8539 (2613) [4134–15 130]	–
Linear acceleration	228 035 (73 244) [79 212–446 257]	247 301 (70 792) [111 594–446 257]	188 813 (62 067) [79 213–360 385]	–
Rotational force	18 285 483 (4 899 556) [3 432 674–31 546 207]	20 283 399 (6 117 025) [8 449 507–44 072 194]	14 218 296 (5 018 048) [6 053 874–28 703 488]	–
Traumatic encephalopathy syndrome^_b_^				
Traumatic encephalopathy syndrome diagnosis, *n* (%)	108 (63%)	77 (67%)	31 (55%)	0 (0%)
Subcategory: cognitive impairment, *n* (%)	98 (57%)	73 (64%)	25 (44%)	5 (9%)
Subcategory: neurobehavioural dysregulation, *n* (%)	97 (57%)	63 (55%)	34 (61%)	1 (1.8%)
Subcategory: cognitive impairment and neurobehavioural dysregulation, *n* (%)	67 (39%)	48 (42%)	19 (34%)	1 (1.8%)

Overview of cohort characteristics including demographics of 170 former football players and 54 unexposed asymptomatic control participants. Values represent mean (standard deviation) [range]. BMI = body mass index; RHI = repetitive head impacts.

^a^Apolipoprotein 4 carrier analysis was only available for 210 participants.

^b^Traumatic encephalopathy syndrome diagnosis and subcategories were completed on all participants.

See Supplementary Table 1 for dichotomized demographics. Note that when we use the terms ‘former American football players’ or ‘former players’, we refer to the combined group of former college and professional players. Dichotomized groups are always identified as either former professional players or former college players.

### Sample characteristics

Data collection for demographics, medical history and athletic history was performed via semi-structured interviews and online questionnaires. Age was collected as a continuous variable. Education was collected in total years. Race and ethnicity were self-reported by participants following a question asking ‘What do you consider your race?’ Participants were then given the following options: American Indian or Alaska Native, Asian Black or African American, Native Hawaiian or other Pacific Islander or White. Participants were additionally asked ‘Do you consider yourself to be either Hispanic or Latino?’ Options included Hispanic or Latino or Not Hispanic or Latino. Participants could select more than one race or ethnicity and were also allowed to refuse to answer or to indicate ‘unknown’. Body mass index (BMI) was calculated using the participant’s height and weight. An aliquot of whole blood was collected from each participant for APOE genotyping (see Table 1 for all cohort characteristics).

### Magnetic resonance imaging

#### Image acquisition

All participants underwent a head MRI at one of the four study imaging sites (Brigham and Women’s Hospital, NYU Langone Medical Center, Cleveland Clinic Lou Ruvo Center for Brain Health in Las Vegas and Mayo Clinic Arizona). All scans followed the same multi-sequence neuroimaging protocol and used the same 3 T scanner model (Siemens Magnetom Skyra; software version VE11), with a 20-channel head coil across the four sites. Relevant to this study was the high resolution (1 × 1 × 1 mm^3^) 3D T1-weighted magnetization prepared rapid gradient echo (MPRAGE) sequence [inversion time = 1100 ms, repetition time (TR) = 2530 ms, echo time (TE) = 3.36 ms, 7° flip angle, 256 field of view (FOV)] and the high resolution (1 × 1 × 1 mm^3^) 3D T2-weighted sampling perfection with application-optimized contrasts by using flip angle evolution (SPACE) (TR = 3200 ms, TE = 412 ms, 256 FOV).

#### Image processing and calculation of cortical thickness and volume

The raw images were visually inspected for completeness, distortion and motion artefacts using 3D Slicer (http://www.slicer.org; version 4.10 Surgical Planning Laboratory Brigham and Women’s Hospital, Boston, MA). Brain masking was performed for all T1-weighted and T2-weighted scans using custom tools developed by the Psychiatry Neuroimaging Laboratory^26,27^ and further processed with FreeSurfer v7.1 to generate cortical and volumetric parcellations according to the Desikan–Killiany atlas.^28-35^ Additionally, whole hippocampus amygdala and hypothalamus volumetric measures were calculated separately using recon_all_hippocampal_subfields_T1T2^36^ and FreeSurfer v7.2 mri_segment_hypothalamic_subunits.^37^ Cortical thickness and volume measures were obtained from the FreeSurfer output.

### Florbetapir PET

PET data were collected at one of the four study imaging sites (Brigham and Women’s Hospital, NYU Langone Medical Center, Cleveland Clinic Lou Ruvo Center for Brain Health in Las Vegas or Banner Alzheimer’s Institute). PET measurements of amyloid-β (Aβ) plaque deposition were acquired using a 370 MBq (10 mCi) bolus injection of florbetapir, a 50-min radiotracer uptake period and a 15-min dynamic emission scan consisting of three 5-min frames.^25,38^ Mean cortical-to-whole cerebellar standard uptake value ratios (SUVRs) and corresponding centiloid values were calculated as previously described.^38-41^ SUVRs ≥ 1.10 (corresponding to centiloid values ≥24.3) have been shown in antemortem PET/post-mortem neuropathological studies to reflect at least moderately frequent neuritic amyloid plaques, a cardinal neuropathological feature of Alzheimer’s disease.^42^

### Exposure to repetitive head injury

Total years in football play were used to assess complete exposure in years starting from youth participation leading to either college or professional play. We additionally evaluated the age of first exposure to assess the impact of early participation in organized tackle football.^21,22,43-45^ Cumulative head impact index scores, including frequency (cumulative hits), linear acceleration and rotational force were estimated based on the self-reported number of seasons of American football played, player position at each career stage and helmet accelerometer data from college players.^46^ Higher cumulative head impact index scores reflect greater estimated exposure to RHIs (see Table 1 for summaries).

### TES diagnosis evaluation of core clinical features and provisional levels of certainty for CTE pathology

All participants were diagnosed through a multidisciplinary diagnostic consensus conference using the NINDS Consensus Diagnostic Criteria for TES.^17^ Consensus conference panelists were presented with the participant’s medical (including neurologic and psychiatric) history; football and other RHI exposure; self- and informant-reported complaints of cognitive, mood and/or behaviour problems, as well as functional dependence status; neurological/motor evaluation findings; and standardized neuropsychological and neuropsychiatric test results. Results of MRI, PET or potential fluid biomarkers were not presented. Based on this information, the panelists used the TES criteria to: (i) confirm substantial exposure to RHI; (ii) evaluate core clinical features involving cognitive impairment (yes/no), neurobehavioural dysregulation (yes/no) and evidence of progressive worsening of clinical symptoms (yes/no); (iii) ascertain whether these core clinical features could be fully accounted for by other disorders; (iv) adjudicate a diagnosis of TES (yes/no) based on information from steps i–iii; (v) grade the level of functional dependence/dementia; (vi) assess the presence of several ‘supportive features’; and (vii) further determine provisional levels of certainty for CTE pathology (suggestive/possible/probable).

Cognitive impairment (yes/no) was evaluated based on four criteria: (i) self-, informant- or clinician-reported cognitive impairment; (ii) significant decline from self-reported former baseline functioning; (iii) impairments in episodic memory and/or executive functioning; and (iv) below 1.5 standard deviations from expected norms on formal neuropsychological testing.^17^ Neurobehavioural dysregulation (yes/no) was evaluated based on these criteria: (i) self-, informant- or clinician-reported neurobehavioural dysregulation; (ii) significant decline from self-reported former baseline functioning; and (iii) symptoms and/or observed behaviours representing poor regulation or control of emotions and/or behaviour.^17^ As mentioned above, TES diagnosis (yes/no) requires evidence of progressive worsening of the core clinical features that cannot be accounted for by other disorders.

Provisional levels of certainty for CTE pathology are based on a stepwise assessment conducted in conjunction with TES diagnosis. The assessment is based on RHI exposure, specific clinical features and a set of supportive features. Classification for the provisional levels of certainty for CTE pathology is not meant to be used for clinical diagnostics purposes. See Katz *et al*.^17^ for full details.

In our analysis, we evaluated whether brain morphometry could predict TES diagnosis (yes/no), TES core clinical cognitive impairment (yes/no), TES core clinical neurobehavioural dysregulation (yes/no) and/or the presence of both cognitive impairment and neurobehavioural dysregulation, as we hypothesized that different core clinical features would be associated with different subsets of CTE regions; see Table 1 for details. Finally, we evaluated whether brain morphometry could predict the provisional levels of certainty for CTE pathology (suggestive/possible/probable). For comprehensive details on neuropsychological test performance in our former American football players, we refer the reader to recently published work by Alosco *et al*.^47^

### Objective neuropsychological evaluation

All participants underwent an in-person baseline neuropsychological test battery utilizing standard paper-pencil tests administered by a fully trained examiner.^25^ For a detailed list of the assessed domains specific neuropsychological tests administered and individual test performances, please refer to the article by Alosco *et al*.^47^ In our examination of neuropsychological test performance, we specifically focused on the top three domains—learning and memory, attention and psychomotor speed and executive function—that displayed impairments. We extracted raw values from these three key assessments: Neuropsychological Assessment Battery (NAB) List Learning Long Delay,^48^ Trail Making Test Part A^49^ and Trail Making Test Part B^49^; see Table 2 for a summary of raw data.

**Table 2:** Neuropsychological test performance of former American football players

Domain	Test	Raw mean	Min	Max
Learning and memory	NAB List Learning Long Delay	5.07 (3.03)	0	11
Attention and psychomotor speed	Trail Making Test Part A	31.4 (13.3)	12.2	118
Executive function	Trail Making Test Part B	82.1 (47.1)	29	300

Overview of neuropsychological test performance of former American football players within three domains. NAB = Neuropsychological Assessment Battery.

### Statistical analysis

#### Group differences

To assess comprehensive differences between former American football players and the unexposed asymptomatic control participants in demographic variables, we performed an independent Welch’s *t*-test on continuous variables (age, BMI and education) and a chi-squared test on categorical variables (race and *APOE4* gene status).

#### Selection of regions of interest

While we have access to all brain regions generated by FreeSurfer, we performed statistical analyses only on brain regions selected *a priori* based on the literature on post-mortem CTE pathology, which included up to stage 3 as discussed in the ‘Introduction’ section. Using a smaller subset of brain regions also preserves statistical power and minimizes type 1 errors. We focused on the following regions: (i) frontal lobe: superior frontal gyrus, caudal middle frontal gyrus and rostral middle frontal gyrus; (ii) temporal lobe: entorhinal cortex, parahippocampal gyrus, insula and temporal pole; and (iii) subcortical structures: amygdala, hippocampus and hypothalamus.^1,2,5,6,15,21,24,45,50,51^

#### Group differences and interactions

To obtain proper effect size estimates of exposure on cortical thickness and volume, we used a generalized least squares model. In this model, we controlled for age, BMI, race, education years, imaging site and apolipoprotein ε4 (*APOE4*) allele status. To estimate the variance across regions, we used the residuals from independent multivariable linear regressions with the aforementioned covariate. We selected these covariates as they have either shown to have effects on ageing, imaging analysis or cortical thickness/volume. Additionally, the volume analyses included total intracranial volume as a covariate. Throughout our analyses, we report the *P*-values adjusted for multiple comparisons using the false discovery rate, where any *P*-values <0.05 were considered significant.

Using this generalized least squares model, we tested for differences in thickness and volume between the unexposed asymptomatic control group and the combined group of former football players as well as *post hoc* analysis of the dichotomized dataset (former professional, former college). We tested interactions with age and exposure factors (total years of football played, cumulative head impact index, seasons, lifetime load: frequency, linear acceleration, rotational force). For the analysis of exposure factors, we evaluated only the combined former football player group. Note that thickness analyses were limited to cortical regions, while volumetric analyses included additional subcortical regions of interest (amygdala, hippocampus, hypothalamus).

We also performed a separate linear regression analysis on total grey matter volume and age to identify a general effect of age controlling for all other covariates listed above. All results are reported using 95% confidence intervals (CI) and *P-*values. Graphical illustrations showing group-level differences were created using *ggseg* (https://github.com/ggseg/ggseg).^52^ In the Supplementary material, we share estimates and 95% CI for all FreeSurfer cortical regions and volumetric outputs (35 left hemisphere × 35 right hemisphere); see Supplementary Tables 3 and 4.

#### Possible overlap with brain regions affected in Alzheimer’s disease

Given the clinical overlap between CTE and other tauopathies, we investigated whether imaging data from our sample of former football players showed volume/thickness reductions in areas not associated with CTE pathology but known to be affected in Alzheimer’s disease. We therefore conducted a separate analysis on regions known to be specific to non-CTE neurodegenerative diseases. Here, based on the literature, we selected the inferior parietal lobe^53,54^ and precuneus.^55-58^ As described earlier, we used a generalized least squares model testing only the left and right inferior parietal lobes and the precuneus. This analysis was done separately from the group-level analysis and was intended only to clarify that the group-level differences did not overlap with regions specific to Alzheimer’s disease.

In addition to these analyses, we conducted a separate investigation following the cortical-cerebellar florbetapir SUVR protocol outlined by Stern *et al*.^38^ In our initial group-level analysis, where we compared former American football players to unexposed asymptomatic controls, we excluded participants with an average cortical florbetapir SUVR of ≥1.1 (*n* = 20) or those lacking a reported SUVR value (*n* = 5). This step was taken to rule out possible Alzheimer's disease pathology as the primary factor influencing our results.

#### Control regions not associated with CTE or Alzheimer’s disease

To ascertain that the observed differences are attributable specifically to CTE-related atrophy resulting from prolonged exposure to RHI, we performed a distinct analysis involving regions unaffected by CTE (control regions) or Alzheimer’s disease. Specifically, we chose bilaterally the lateral occipital cuneus and pericalcarine regions.

#### Associations between brain regions and TES diagnosis and level of certainty for CTE pathology

To understand the relationship between brain morphometry and TES diagnosis and core clinical features of cognitive impairment and neurobehavioural dysregulation, we performed a logistic regression analysis to predict each TES outcome from each brain region of interest after controlling for age, race, BMI, education, imaging site, *APOE4* gene status, football status (collegiate or professional) and total intracranial volume. The four TES outcomes included: (i) TES diagnosis (yes/no); (ii) TES core cognitive impairment feature (yes/no); (iii) TES core neurobehavioural dysregulation feature (yes/no); and (iv) both TES core cognitive impairment features (yes/no) and neurobehavioural dysregulation (yes/no).

To understand the relationship between brain morphometry and the provisional levels of certainty for CTE pathology (suggestive/possible/probable), we performed an independent multinomial model to predict each provisional level of certainty for CTE pathology from each brain region. We controlled for age, race, BMI, education, imaging site, *APOE4* gene status, football status (collegiate or professional) and total intracranial volume. The provisional levels of certainty for CTE pathology included: (i) suggestive of CTE; (ii) possible CTE; and (iii) probable CTE. We note that we did not include definite CTE with TES, as no participant in our study met this criterion.

Across both analyses, false discovery rate adjusted *P*-values were calculated to control for multiple testing of the effect of each brain region on each TES outcome and provisional levels of certainty for CTE pathology. These analyses involving TES variables and provisional levels of certainty for CTE pathology only included data from former American football players.

#### Associations between regions of interest and individual neuropsychological test performance

To understand the association between our regions of interest associated with CTE pathology at post-mortem and the individualized raw neuropsychological assessments (NAB List Learning Long Delay, Trail Making Test Part A and Trail Making Test Part B), we performed a regression analysis controlling for age, race, BMI, education, imaging site, *APOE4* gene status and total intracranial volume. In this analysis, we adjusted for multiple comparisons using the Benjamini and Hochberg method. This analysis only included data from former American football players. Two participants were excluded from the analysis for missing data.

## Results

### Demographical factors

Using Welch’s two-sample *t*-test, we identified group-level differences in BMI between our former American football players and unexposed asymptomatic controls [*t*(90.3) = 2.3, mean difference 1.7, (95% CI: 0.2, 3.1), *P* = 0.024], indicating a higher BMI in our football players. No other group differences were observed.

### Group differences and interactions

#### Cortical thickness

**Figure 1:**
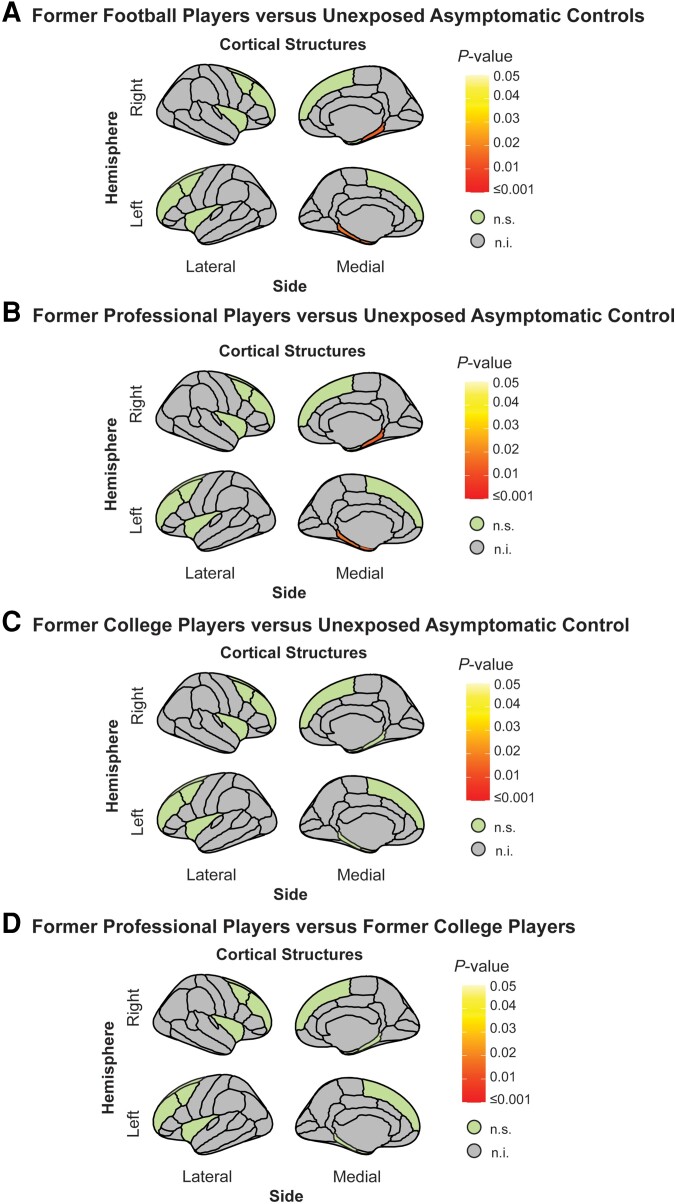
**Cortical thickness group-level differences**. (**A**) Cortical thickness group differences between former American football players (*n* = 170) and unexposed asymptomatic controls (*n* = 54). Results indicate reduced cortical thickness in the left hemisphere: entorhinal cortex and parahippocampal gyrus and right hemisphere: parahippocampal gyrus in our former American football players compared to unexposed asymptomatic controls. (**B**) Subgroup analysis of former professional players (*n* = 114) and unexposed asymptomatic controls (*n* = 54). Results indicate reduced cortical thickness in the left hemisphere: entorhinal cortex and parahippocampal gyrus and right hemisphere: parahippocampal gyrus in our former professional football players compared to unexposed asymptomatic controls. (**C**) Group differences between former college players (*n* = 56) and unexposed asymptomatic controls (*n* = 54). No group-level differences were observed. (**D**) Group differences between former professional players (*n* = 114) and former college players (*n* = 56). No group differences were observed. All reported *P-*values are corrected for multiple comparisons. n.i. = not included; n.s. = not significant.

Using the generalized least squares model, we tested for differences in cortical thickness between the unexposed asymptomatic control group and the combined group of former football players. This model identified two left hemisphere brain regions [entorhinal cortex: (95% CI: −0.2, −0.05) *P* = 0.01; parahippocampal gyrus: (95% CI: −0.16, −0.03), *P* = 0.01] and one right hemisphere brain region [parahippocampal gyrus: (95% CI: −0.16, −0.03), *P* = 0.01], which showed reduced cortical thickness in former American football players compared to unexposed asymptomatic controls; see Fig. 1A.

*Post hoc* analysis dichotomizing the former football player data set into former professional and former college football players identified group-level differences between former professional players and unexposed asymptomatic controls in two left hemisphere regions [entorhinal cortex: (95% CI: −0.23, −0.05), *P* = 0.01; parahippocampal gyrus: (95% CI: −0.1, −0.03), *P* = 0.01] and one right hemisphere region [parahippocampal gyrus: (95% CI: −0.16, −0.03), *P* = 0.016]; see Fig. 1B. No other *post hoc* analysis comparing groups (either former college versus unexposed asymptomatic controls; or former professional versus former college) reached significance (all *P*-values >0.3; see Fig. 1C and D, Table 3 and Supplementary Table 1 for a summary of all findings).

**Table 3:** Group differences for cortical thickness

Group comparison and region of interest	Left				Right			
	Estimate	SD	95% CI	*P*-value	Estimate	SD	95% CI	*P*-value
**Former football player versus unexposed asymptomatic control**								
Superior frontal gyrus	0.01	0.01	−0.02, 0.04	0.6	0.01	0.02	−0.02, 0.05	0.67
Rostral middle frontal gyrus	0.006	0.01	−0.02, 0.03	0.6	0.004	0.01	−0.02, 0.03	0.77
Caudal middle frontal gyrus	−0.02	0.02	−0.05, 0.01	0.4	0.006	0.02	−0.03, 0.04	0.77
Entorhinal cortex	−0.13	0.04	−0.2, −0.05	**0**.**01**	−0.05	0.04	−0.14, −0.03	0.5
Parahippocampal gyrus	−0.1	0.03	−0.16, −0.02	**0**.**01**	−0.1	0.03	−0.16, −0.03	**0**.**01**
Insula sulcus	−0.04	0.02	−0.08, 0.01	0.2	−0.03	0.02	−0.1, 0.01	0.5
Temporal pole	−0.07	0.05	−0.16, −0.02	0.2	−0.04	0.05	−0.13, 0.06	0.67

All *P-*values are corrected for multiple comparisons. All significant values at *P* < 0.05 are in bold. CI = confidence interval; SD = standard deviation.

#### Volume

Using the generalized least squares model, we tested for differences in volume between the unexposed asymptomatic control group and the combined former football players. For reference, our volume analysis included subcortical structures (hippocampus, amygdala and hypothalamus).

**Figure 2:**
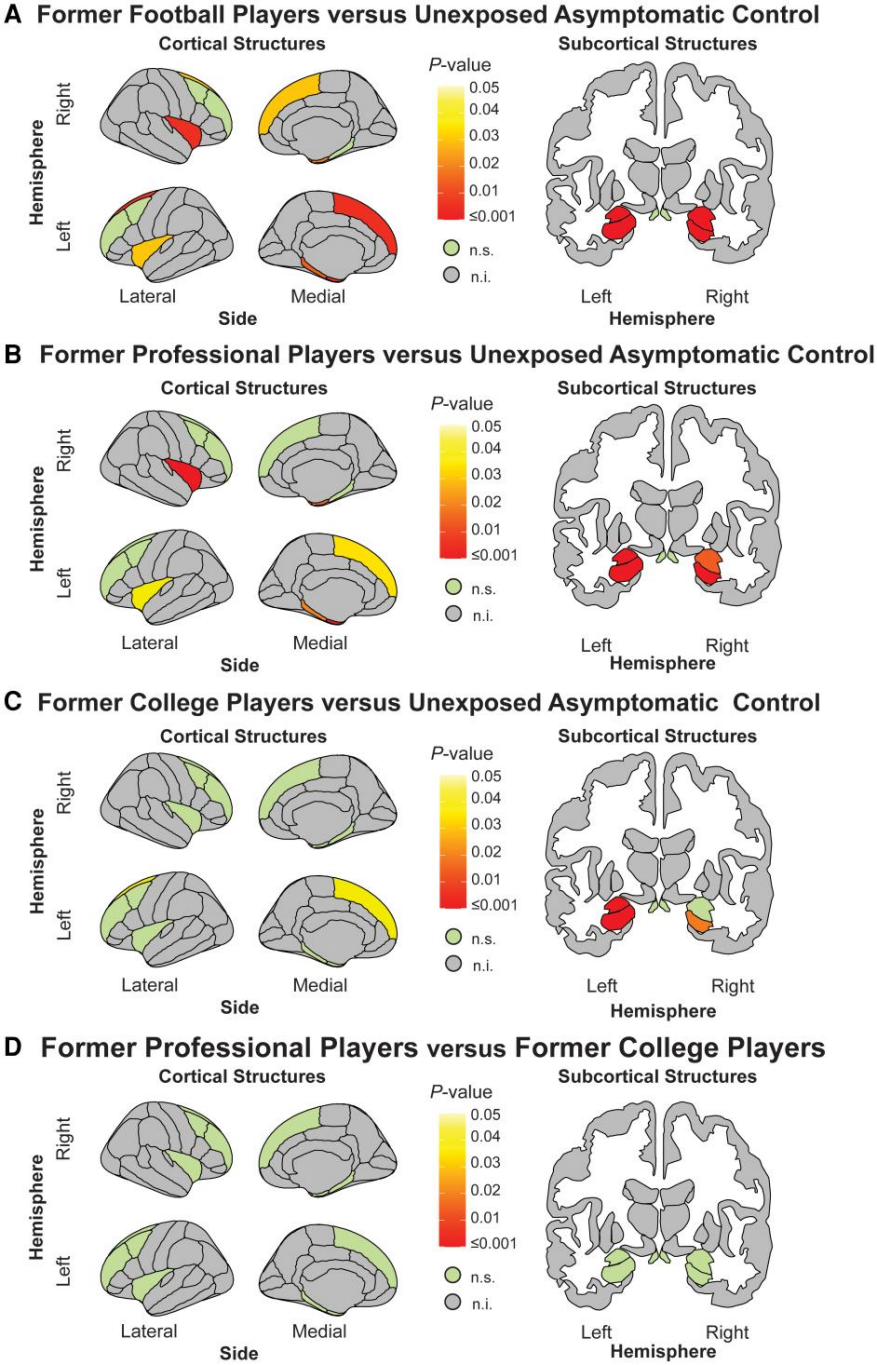
**Volumetric group-level differences**. (**A**) Volumetric group differences between former American football players (*n* = 170) and unexposed asymptomatic controls (*n* = 58). Results indicate reduced volume in the left hemisphere: amygdala, hippocampus, entorhinal cortex, parahippocampal gyrus, insula and superior frontal gyrus; and right hemisphere: amygdala, hippocampus, temporal pole, entorhinal cortex, parahippocampal gyrus, insula and superior frontal gyrus in our former American football players compared to unexposed asymptomatic controls. (**B**) Group differences between former professional players (*n* = 114) and unexposed asymptomatic controls (*n* = 54). Results indicate reduced volume in the left hemisphere: amygdala, hippocampus, entorhinal cortex, parahippocampal gyrus, insula and superior frontal gyrus; and right hemisphere: amygdala, hippocampus, entorhinal cortex, insula and temporal pole in our former professional players compared to unexposed asymptomatic controls. (**C**) Group differences between former college players (*n* = 56) and unexposed asymptomatic controls (*n* = 54). Results indicate reduced volume in the left hemisphere: amygdala, hippocampus and superior frontal gyrus; and right hemisphere: hippocampus in our former professional players compared to unexposed asymptomatic controls. (**D**) Group differences between former professional players (*n* = 114) and former college players (*n* = 54). No group differences were observed. All reported *P-*values are corrected for multiple comparisons. n.i. = not included; n.s. = not significant.

**Figure 3:**
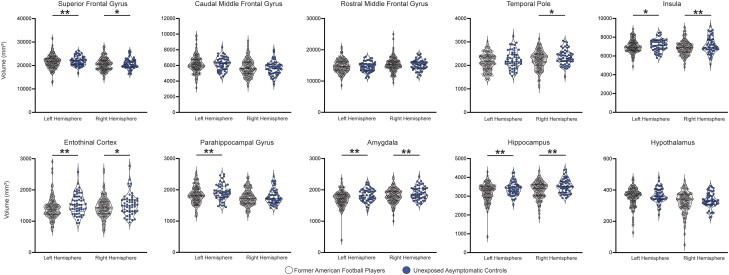
**Raw volumetric data for preselected chronic traumatic encephalopathy (CTE)-related regions in former American football players and unexposed asymptomatic controls**. Violin plots showing the preselected CTE-related regions and individual raw data-points. Significant group-level differences are indicative of our volumetric main group-level result of former American football players and unexposed asymptomatic controls. Significant differences are observed in the superior frontal gyrus, temporal pole, insula, entorhinal cortex, parahippocampal gyrus, amygdala and hippocampus. Outer horizontal lines indicate the interquartile range and the middle line indicates the median. **P ≤ 0.01, *P < 0.05. *P*-values are corrected for multiple comparisons.

The volume analysis identified six left hemisphere brain regions [hippocampus: (95% CI: −334, −94.5), *P* < 0.01; amygdala: (95% CI: −184.5, −46), *P* < 0.01; entorhinal cortex: (95% CI: −258.6, −.68), *P* < 0.01; superior frontal gyrus: (95% CI: −1479, −331), *P* < 0.01; parahippocampal gyrus: (95% CI: −197, 33), *P* = 0.01; insula: (95% CI: −404, −35), *P* = 0.03] and six right hemisphere brain regions [hippocampus: (95% CI: −352, −108), *P* < 0.01; amygdala: (95% CI: −162.5, −38), *P* < 0.01; insula: (95% CI: 610, −170), *P* < 0.01; temporal pole: (95% CI: −254, −31.5), *P* = 0.02; entorhinal cortex: (95% CI: −247.6, −36.5), *P* = 0.02; superior frontal gyrus: (95% CI: −1296.7, −93.6), *P* = 0.03], showing reduced volume in former football players compared to unexposed asymptomatic controls; Figs. 2A and 3. Two additional regions showed borderline significance [left hemisphere caudal middle frontal gyrus: (95% CI: −488, −3.5), *P* = 0.068; right parahippocampal gyrus: (95% CI: −156, −2.2), *P* = 0.07]; see Fig. 3 for full details.

Following this, a *post hoc* analysis dichotomizing the former football player group (former professional former college) showed reduced volume in former professional players compared to unexposed asymptomatic controls in six left hemisphere brain regions [entorhinal cortex: (95% CI: −299, −97), *P* < 0.001; hippocampus: (95% CI: −340, −80), *P* < 0.01; amygdala: (95% CI: −186, −35), *P* < 0.01; parahippocampal gyrus: (95% CI: −190, 28), *P* = 0.02; insula: (95% CI: −401, −34.6), *P* = 0.04); superior frontal gyrus: (95% CI: −1327, −132), *P* < 0.03] and five right hemisphere brain regions [hippocampus: (95% CI: −398.5, −117), *P* < 0.01; amygdala: (95% CI: −188, −147.5), *P* = 0.02; insula: (95% CI: 658, −147.5), *P* < 0.01; temporal pole: (95% CI: −286, −39), *P* = 0.02; entorhinal cortex: (95% CI: −282, −42), *P* = 0.02]; see Fig. 2B. *Post hoc* analysis comparing group-level differences between former college and unexposed asymptomatic controls identified three left hemisphere brain regions [hippocampus: (95% CI: −508, −141), *P* < 0.01; amygdala: (95% CI: −258, −65), *P* < 0.01; superior frontal gyrus: (95% CI: −1908, −264), *P* = 0.04] and one right hemisphere brain region [hippocampus: (95% CI: −420.5, −83), *P* = 0.02] indicating reduced volume in the former college players compared to the unexposed asymptomatic controls; see Fig. 2C. No other *post hoc* analyses comparing groups (former professional versus former college) reached statistical significance (all *P*-values >0.3; see Fig. 2D, Table 4 and Supplementary Table 2 for a summary of all findings).

**Table 4:** Group differences for volume

Group comparison and region of interest	Left				Right			
	Estimate	SD	95% CI	*P*-value	Estimate	SD	95% CI	*P*-value
**Former football player versus unexposed asymptomatic control**								
Superior frontal gyrus	−919	300	−1479, −331	**<0**.**01**	−728	308	−1296, −93	**0**.**03**
Rostral middle frontal gyrus	−188	243	−679, 272	0.5	−208	260	−753, 303	0.5
Caudal middle frontal gyrus	−242	122	−488, 3	0.067	−94	149	−398, 191	0.6
Entorhinal cortex	−16	50	−258, −68	**<0**.**01**	−139	53	−247, −36	**0**.**02**
Parahippocampal gyrus	−110	41	197, −33	**0**.**01**	−76	39	−156, −2	0.07
Insula sulcus	−220	93	−404, −34	**0**.**03**	−386	117	−610, −170	**<0**.**01**
Temporal pole	−105	64	−230, −15	0.1	−156	56	−254, −31	**0**.**02**
Amygdala	−119	36	−184, −46	**<0**.**01**	−98	32	−162, −39	**<0**.**01**
Hippocampus	−220	63	−334, 94	**<0**.**01**	−226	62	−352, −108	**<0**.**01**
Hypothalamus	−5	8	−21, 10	0.5	1	8	−14, 16	0.87

All *P-*values are corrected for multiple comparisons. Volume analysis includes subcortical regions. All significant values at *P* < 0.05 are in bold. CI = confidence interval; SD = standard deviation.

#### Interactions with age and exposure

We did not find an Age × Group interaction for either cortical thickness or volume within the preselected regions of interest (all *P*-values >0.07). Although while analysing the total grey matter, we noted a main effect of age in both former American football players (*F* = −3188, *P* < 0.00001) and unexposed asymptomatic controls (*F* = −1702, *P* = 0.01). This aligned with the expected age-related changes seen in total grey matter volume.

We found a significant association between the volume of the right insula and the age of first exposure [(95% CI: 18, 89), *P* = 0.03] but interactions between the volume or cortical thickness of the other preselected regions of interest and age of first exposure were not significant (all *P*-values >0.057). Similarly, neither cortical thickness nor volume was associated with total years of football or cumulative head impact indices (frequency–cumulative hits linear acceleration or rotational force) within the former football player group (all *P*-values >0.4).

To understand the effects of exposure factors (total years of football and cumulative head impact indexes of frequency, linear acceleration and rotational force) in former professional players given their extensive participation in contact sports we conducted a secondary analysis solely within this group. We again found that neither reduced cortical thickness nor reduced volume was associated with total years of football or cumulative head impact index, frequency (cumulative hits) linear acceleration and rotational force (all *P*-values >0.16).

#### Possible overlap with brain regions affected in Alzheimer’s disease

We did not find group-level differences between the former American football players and unexposed asymptomatic controls in both cortical thickness and volume within our specific brain regions commonly associated with Alzheimer’s disease pathology but not CTE (all *P*-values >0.26).

Next, when removing participants with a florbetapir SUVR of ≥1.1, our findings remained consistent across cortical thickness and volume. In cortical thickness, our analysis identified one left hemisphere brain region [entorhinal cortex: (95% CI: −0.2, −0.04) *P* = 0.03] and one right hemisphere brain region [parahippocampal gyrus: (95% CI: −0.16, −0.04) *P* = 0.01], which showed reduced cortical thickness in former American football players compared to unexposed asymptomatic controls.

The volume analysis identified six left hemisphere brain regions [hippocampus: (95% CI: −357, −113), *P* < 0.01; amygdala: (95% CI: −194, −49), *P* < 0.01; entorhinal cortex: (95% CI: −285, −76), *P* < 0.01; superior frontal gyrus: (95% CI: −1541, −326), *P* < 0.01; parahippocampal gyrus: (95% CI: −189, 17), *P* = 0.04; insula: (95% CI: −436, −77), *P* = 0.01] and seven right hemisphere brain regions [hippocampus: (95% CI: −376, −130), *P* < 0.001; amygdala: (95% CI: −185, −44), *P* < 0.01; insula: (95% CI: 700.5, −200), *P* < 0.001; temporal pole: (95% CI: −248, −13), *P* = 0.04; entorhinal cortex: (95% CI: −279, −42), *P* = 0.02; superior frontal gyrus: (95% CI: −1385, −188.5), *P* = 0.02 parahippocampal gyrus: (95% CI: −173, −5.5), *P* = 0.04] showed reduced volume in former football players compared to unexposed asymptomatic controls. These results are similar to those of our main group findings.

#### Control regions not associated with CTE or Alzheimer’s disease

We did not find group-level differences between the former American football players and unexposed asymptomatic controls in both cortical thickness and volume within our pre-selected control regions (all *P*-values >0.07).

#### Associations between brain regions and TES diagnosis and level of certainty for CTE pathology

No associations between the preselected brain regions of interest and the four TES outcomes (e.g. TES diagnosis TES cognitive impairment TES neurobehavioural dysregulation and both TES cognitive impairment and neurobehavioural dysregulation) were significant (all *P*-values >0.2). Additionally, no associations between the preselected brain regions of interest and the provisional levels of certainty for CTE pathology (e.g. suggestive/possible/probable) were significant (all *P*-values >0.1).

#### Associations between regions of interest and individual neuropsychological test performance

The regression analysis revealed two notable associations between the preselected CTE-related pathology brain regions of cortical thickness and the neuropsychological assessments. First in the left hemisphere superior frontal gyrus [(95% CI: −0.0012, −0.0004), *P* = 0.01] and correlated with Trail Making Test Part B. Lastly, an association between left hemisphere insula [(95% CI: −0.0014, −0.0004) *P* = 0.01] with Trail Making Test Part B. Within both cases, worse performance was associated with decreased cortical thickness.

In brain volume, the regression analysis revealed four notable associations. These associations include the superior frontal gyrus in both left [(95% CI: −22.6, −8), *P* = 0.003] and right [(95% CI: −21, −7.2), *P* = 0.003] hemispheres linked to Trail Making Test Part B the caudal middle frontal gyrus in the left [(95% CI: −8.7 −2.4), *P* = 0.01] hemisphere associated with Trail Making Test Part B and the superior frontal gyrus in the left [(95% CI: −39, −64.4), *P* = 0.04] hemisphere correlated with Trail Making Test Part A. Within all associations worse performance was associated with decreased volume to its corresponding region.

## Discussion

Overall, our main findings showed reduced *in vivo* cortical thickness and cortical/subcortical volume in former American football players compared to same-age men without exposure to football or other contact sports or a history of TBI in several brain regions that are similar to those impacted by CTE pathology as observed in post-mortem pathology studies.^2-5,11^ We found an association between the age of first exposure and the volume of the right insula although we did not observe associations between brain morphometry in any other CTE regions or exposure metrics (age of first exposure, total years of football played or cumulative head impact indices). Additionally, we found an association between age and total grey matter volume loss in our former football group and our control group. However, Age × Group interactions at the region of interest level did not reveal any significant relationships. We did not observe any interactions between brain morphometry and TES diagnosis the core clinical features or the provisional levels of certainty for CTE pathology.

### Thickness and volume reduction in former American football players

Former American football players showed reduced volume compared to unexposed asymptomatic controls in the superior frontal gyrus entorhinal cortex parahippocampal gyrus insula temporal pole amygdala and hippocampus. Additionally, *post hoc* analyses indicated that compared to unexposed asymptomatic controls former professional players had reduced volume in six regions (entorhinal cortex, parahippocampal gyrus, insula, superior frontal gyrus, amygdala and hippocampus), while former college players showed only three regions with reduced volume (superior frontal gyrus, amygdala and hippocampus). This finding suggested that the level of exposure and intensity of play may negatively impact brain morphometry. Note however that we did not observe group-level differences when directly comparing former college to former professional players.

Our findings revealed that post-mortem CTE neuropathological-related changes can be observed *in vivo* in this population suggesting further that structural MRI is a valuable tool to characterize the long-term consequences of exposure to RHI. Importantly we did not find morphometric differences in regions known to be affected in Alzheimer’s disease but not observed post-mortem in CTE (i.e. inferior parietal lobe^53,54^ and precuneus^55-58^). Moreover, in our follow-up analysis, excluding participants with a PET florbetapir SUVR of ≥1.1 indicative of moderate-to-frequent neuritic Aβ plaques primarily observed in Alzheimer’s disease, our primary group results remained unchanged. This suggested that our findings may not be exclusively linked to Alzheimer’s disease. However, we note that other related dementias should be ruled out as well. This warrants future studies to investigate vascular dementias and the role of cerebrovascular pathology and its impact on brain structure. In our sample, cardiovascular risk profiles were complex, with former American football players having higher BMI and higher prevalence of sleep apnoea but a lower stroke risk and lower blood pressure compared to the unexposed asymptomatic controls.^59^ This highlights the need for future investigations into the underlying mechanisms and potential implications for brain structure. Finally in a subsequent analysis to ensure that our results were specific to CTE-related atrophy resulting from prolonged exposure to RHI, we tested regions unrelated to both CTE and Alzheimer’s disease pathology (lateral occipital cuneus and pericalcarine regions). We found no significant results within these control regions indicating that our findings may be specific to CTE-related atrophy resulting from prolonged exposure to RHI.

A strength of this study is the relatively large sample size; DIAGNOSE CTE has the largest sample of *in vivo* structural MRI data from former professional players (*n* = 114 versus *n* < 75 in recent studies^45,60-62^) and is the only study that also includes a sample from former college players (*n* = 56). Most brain morphometry studies with large sample sizes have focused on young active college or high school American football players.^63-67^ Additionally, no study has focused on the potential for connecting *in vivo* MRI with established post-mortem morphometric observations in CTE, although many found reduced volume in regions overlapping with our findings, especially the hippocampus.^10,11,21,23,24,43-45,60,68-74^

### Association between brain volume and age

We observed a negative association between age and total grey matter volume in the former players and the unexposed asymptomatic control group. Nevertheless, our results align with prior studies indicating age-related effects on brain morphometry in both controls and athletes.^75-80^ These results suggested that former players may experience accelerated volume reduction with age like findings from studies in other neurodegenerative diseases.^81-85^ Note that we did not observe an Age × Group (former players versus unexposed asymptomatic controls) interaction in regions specific to CTE pathology, and further studies are needed to confirm accelerated volume reduction in former players and identify regions most likely to be affected.

### Brain volume exposure TES diagnosis core clinical features and provisional levels of certainty for CTE pathology

In the former American football group, we observed one association between the right insular volume and the age of first exposure where a smaller volume is associated with an earlier age of first exposure. This finding was moderated by the lack of observed interactions between any other morphometric measures and exposure factors (age of first exposure, total years of football, cumulative head impact index measures). Furthermore, we did not observe an association between volume and TES diagnosis core clinical features or the provisional levels of certainty for CTE pathology.

These results were unexpected, as previous studies in former American football players have found more robust associations between brain morphometry and exposure metrics such as the age of first exposure to RHI.^21,45^ Additionally, the age of first exposure has been reported to be influential as a factor in determining which former American football players exposed to extensive RHIs develop cognitive impairment and neurobehavioural changes later in life.^22,43,70,73,86,87^ It may be that the regions of interest we selected were impacted by a binary measure of exposure (yes/no) as demonstrated by our group comparison between players and unexposed asymptomatic control but that continuous measures of exposure in football players are not as strongly associated with volume reduction. In other words, we may have observed a ceiling effect whereby, beyond a specific threshold, more exposure does not further influence the region of interest volume reduction. This needs to be elucidated in future studies with potentially different exposure measures and/or selection of regions of interest.

Concerning TES diagnosis the core clinical features of cognitive deficit and neurobehavioural dysregulation and the provisional levels of certainty for CTE pathology, our negative findings indicate a disconnect between the consensus of TES criteria and brain morphometry. Even though we found morphometric differences between former players and controls, volume and cortical thickness alone are not strong predictors of TES, at least not in the regions we selected. This study adds to the limited specificity of CTE pathology within the TES consensus criteria that has been reported by others and has now been shown in the provisional levels of certainty for CTE pathology, which is also solely based on the level of clinical symptom severity.^18^ Future studies with other regions of interest or MRI modalities (such as diffusion or functional MRI) are needed to further explore this relationship. Additionally, the TES criteria have not been validated by post-mortem examination; such studies may lead to TES revisions and better associations between TES and MRI measures.

Moreover, within the former American football players, we identified four correlations between individualized neuropsychological test performance assessed through raw scores and our predetermined CTE-related pathology regions. These associations were predominantly observed in two distinct domains—attention and psychomotor speed, and executive function—signifying poorer performance with decreased brain volume. While we couldn't establish associations with TES diagnosis or the core clinical features of cognitive deficit and neurobehavioural dysregulation, we were able to identify correlations in individualized test performance highlighting the most substantial impairments as reported in the full neuropsychological test performance of our sample.^47^ We therefore suggest that future studies should consider evaluating individualized neuropsychological test performance.

### Limitations

While it is important to note the subject sample from the DIAGNOSE CTE Research Project is the largest cohort to date it has limitations. The participants consisted only of self-identified males who played American football at all levels (youth–professional) between 1952 and 2007. This limits the generalizability of our findings, as the sport of American football has evolved rapidly both in its intensity of play and its health and safety protocols. We also cannot directly infer from this study the impact of RHI in other sports or in other genders. Importantly, we acknowledge that our unexposed asymptomatic control participants were all asymptomatic at the time of screening, which may impact our group-level comparisons. Although a relatively large percentage of the former professional players (42%) and unexposed controls (40%) identify as being Black (similar to the approximately 40% proportion of Black former NFL players who played between 1967–1996, the years our sample would have played), our former college players are younger and include more individuals who identify as White.

In terms of methodology, a further limitation was that the cumulative head impact index scores were not derived from helmet accelerometer data from professional football players, as no such data are available for this sample. Rather, they were estimated based on the self-reported number of seasons of American football played, player position at each level, and helmet accelerometer data from youth high school and collegiate athletes. Furthermore, some regions known to be affected by CTE post-mortem could not confidently be extracted *in vivo* using FreeSurfer, given the typical overestimates misidentifying structures surrounding high-intensity voxels (e.g. substantia nigra, mammillary bodies, midbrain structures, cerebellar regions). Additionally, while we showed that our findings are unlikely related to Alzheimer’s disease, future studies should include other related dementias to confirm that the regions we identified are indeed most likely explained by a CTE pathology. However, a major limitation of our work was that we do not have post-mortem data for our participants to determine the underlying pathology. Finally, our longitudinal neuroimaging design was interrupted by the coronavirus disease 2019 pandemic. Therefore, we were not able to evaluate disease progression.

## Conclusions

In summary, this study reports reduced cortical thickness and volume in former American football players compared to unexposed asymptomatic controls in regions known to be affected by CTE at post-mortem. This confirms that findings consistent with post-mortem pathology are observable *in vivo* in this population. Contrary to our initial hypotheses, we did not observe strong interactions between morphometric measures and exposure metrics or TES diagnosis and core clinical features. These findings need to be further investigated and future research should aim at understanding what factors predict a higher probability of developing CTE in those extensively exposed to RHIs.

## Supplementary Material

awae098_Supplementary_Data

## Data Availability

Data from the DIAGNOSE CTE Research Project will be available to qualified investigators through the Federal Interagency Traumatic Brain Injury Research (FITBIR) Informatics System through the National Institutes of Health (NIH) Center for Information Technology: https://fitbir.nih.gov/content/access-data. DIAGNOSE CTE Research Project data including those reported in this study will also be available to qualified investigators through a project-specific data-sharing portal. Interested investigators should contact Dr Robert A. Stern, bobstern@bu.edu.
