# Supplementary material for: Brain morphometry in former American football players: findings from the DIAGNOSE CTE research project
Source: Brain. 2024 Mar 27;147(10):3596–610. doi: 10.1093/brain/awae098 (PMC11449133; doi:10.1093/brain/awae098)
Supplement: awae098_Supplementary_Data [file awae098_supplementary_data.pdf]

Group Comparison & Region of Interest	Left				Right			
	Estimate	SD	95% CI	P Value	Estimate	SD	95% CI	P Value
**Former Professional Players vs Unexposed Asymptomatic Control**								
Superior Frontal Gyrus	.01	.02	[-.02, .05]	.5	.02	.02	[-.02, .05]	.6
Rostral Middle Frontal Gyrus	.01	.01	[-.02, .03]	.6	.01	.01	[-.02, .04]	.7
Caudal Middle Frontal Gyrus	-.02	.02	[-.06, .01]	.35	-.0005	.02	[-.04, .04]	.97
Entorhinal Cortex	-.1	.05	[-.2, -.05]	**.01**	-.06	.05	[-.16, -.02]	.6
Parahippocampal Gyrus	-.1	.03	[-.16, -.03]	**.01**	-.1	.03	[-.16, -.03]	**.017**
Insula Sulcus	-.03	.02	[-.08, .02]	.35	-.03	.03	[-.1, .02]	.6
Temporal Pole	-.05	.05	[-.1, .05]	.37	-.04	.05	[-.1, .07]	.6
**Former College Players vs Unexposed Asymptomatic Control**								
Superior Frontal Gyrus	.0004	.02	[-.04, .05]	.98	.01	.02	[-.03, .05]	.97
Rostral Middle Frontal Gyrus	.01	.02	[-.05, .04]	.7	.0006	.02	[-.03, .04]	.97
Caudal Middle Frontal Gyrus	-.003	.02	[-.05, .04]	.98	.02	.02	[-.02, .07]	.97
Entorhinal Cortex	-.1	.06	[-.1, .01]	.3	-.006	.05	[-.1, .1]	.97
Parahippocampal Gyrus	-.06	.04	[-.1, .01]	.3	-.07	.03	[-.1, .0002]	.3
Insula Sulcus	-.03	.03	[-.1, .04]	.7	-.02	.03	[-.1, .05]	.97
Temporal Pole	.1	.07	[-.2, .03]	.3	-.02	.06	[-.03, .04]	.97
**Former College Players vs Former Professional Players**								
Superior Frontal Gyrus	.01	.02	[-.03, .05]	.8	.01	.02	[-.02, .05]	.9
Rostral Middle Frontal Gyrus	-.004	.02	[-.03, .03]	.8	.02	.01	[-.01, .05]	.9
Caudal Middle Frontal Gyrus	-.02	.02	[-.06, .02]	.6	-.001	.02	[-.05, .04]	.9
Entorhinal Cortex	.02	.04	[-.1, .1]	.8	-.004	.03	[-.08, .07]	.9
Parahippocampal Gyrus	-.04	.03	[-.1, 03]	.6	-.01	.03	[-.07, 04]	.9
Insula Sulcus	.01	.02	[-.03.07]	.8	.03	.02	[-.02.08]	.9
Temporal Pole	.1	.06	[-.01, .2]	.5	-.02	.05	[-.1, .1]	.9

Supplementary Table 1. Group differences for cortical thickness. All p values are corrected for multiple comparisons.
*All significant values at p <.05 are bolded.

Group Comparison & Region of Interest	Left				Right			
	Estimate	SD	95% CI	P Value	Estimate	SD	95% CI	P Value
**Former Professional Players vs Unexposed Asymptomatic Control**								
Superior Frontal Gyrus	-743	313	[-1327, -132]	**.03**	-621	331	[-1258, -36]	.1
Rostral Middle Frontal Gyrus	-129	248	[-623, 356]	.7	-59	303	[-647, 538]	.8
Caudal Middle Frontal Gyrus	-225	95	[-507, 29]	.1	-208	163	[-517, 94]	.2
Entorhinal Cortex	-195	51	[-299, -97]	**<.01**	-156	60	[-282, -42]	**.02**
Parahippocampal Gyrus	-109	42	[-190, -28]	**.02**	-71	43	[-159, 13]	.1
Insula Sulcus	-214	95	[-401, -35]	**.04**	-410	132	[-398, -117]	**<.01**
Temporal Pole	-91	65	[-216, 35]	.2	-160	62	[-286, -39]	**.02**
Amygdala	-111	37	[-186, -35]	**<.01**	-107	39	[-188, -35]	**.02**
Hippocampus	-212	66	[-340, -80]	**<.01**	-252	72	[-498, -117]	**<.01**
Hypothalamus	-1	9	[-17, 17]	.9	3	9	[-14, 20]	.8
**Former College Players vs Unexposed Asymptomatic Control**								
Superior Frontal Gyrus	-1073	430	[-1907, -264]	**.04**	-702	411	[-1506, 68]	.2
Rostral Middle Frontal Gyrus	-333	389	[-1115, 426]	.4	-364	390	[-1111, 412]	.4
Caudal Middle Frontal Gyrus	-158	171	[-501, 203]	.4	249	182	[-127, 604]	.3
Entorhinal Cortex	-55	69	[-194, 75]	.4	-54	67	[-181, 69]	.46
Parahippocampal Gyrus	-74	52	[-174, 33]	.3	-49	49	[-144, 46]	.4
Insula Sulcus	-170	137	[437, 94]	.3	-274	154	[568, 38]	.2
Temporal Pole	-128	90	[-294, 51]	.3	-113	71	[-254, 24]	.2
Amygdala	-158	50	[-258, -65]	**<.01**	-98	42	[-184, -18]	.1
Hippocampus	-321	93	[-508, -141]	**<.01**	-257	84	[-420, -83]	**.02**
Hypothalamus	-15	11	[-38, 7]	.3	-4	13	[-31, 22]	.8
**Former College Players vs Former Professional Players**								
Superior Frontal Gyrus	400	338	[-256, 1061]	.6	339	325	[-279, 971]	.6
Rostral Middle Frontal Gyrus	193	296	[428, 750]	.7	657	345	[-21, 1317]	.3
Caudal Middle Frontal Gyrus	39	180	[-327, 408]	.8	-334	158	[-632, -41]	.3
Entorhinal Cortex	-78	53	[188, 25]	.6	-53	53	[-154, 52]	.6
Parahippocampal Gyrus	-63	44	[-143, 22]	.6	-23	40	[-100, 59]	.7
Insula Sulcus	-61	98	[-247, 131]	.7	-110	112	[-328, 117]	.6
Temporal Pole	46	72	[-97, 185]	.7	-49	67	[-184, 83]	.7
Amygdala	10	43	[-73, 99]	.8	-23	41	[-107, 56]	.7
Hippocampus	35	84	[-125, 200]	.8	-12	85	[-181, 156]	.9
Hypothalamus	16	12	[-6, 41]	.6	5	13	[-19, 32]	.8

Supplementary Table 2. Group differences for volume. All p values are corrected for multiple comparisons. Volume analysis includes subcortical regions.
*All significant values at p <.05 are bolded.

Region of Interest	Left			Right		
	Estimate	SD	95% CI	Estimate	SD	95% CI
Superior Frontal Gyrus	0.01	0.02	[-0.02, 0.04]	0.01	0.02	[-0.02, 0.05]
Rostral Middle Frontal Gyrus	0.01	0.01	[-0.02, 0.03]	0.004	0.02	[-0.03, 0.03]
Caudal Middle Frontal Gyrus	-0.02	0.02	[-0.05, 0.01]	0.01	0.02	[-0.03, 0.04]
Pars Opercularis	-0.04	0.02	[-0.07, -0.001]	-0.05	0.02	[-0.09, -0.01]
Pars Triangularis	-0.01	0.02	[-0.04, 0.02]	0.01	0.02	[-0.02, 0.05]
Pars Orbitalis	-0.03	0.02	[-0.08, 0.01]	-0.01	0.02	[-0.05, 0.03]
Lateral Orbital Frontal Cortex	-0.02	0.02	[-0.06, 0.01]	-0.001	0.02	[-0.04, 0.04]
Medial Orbital Frontal Cortex	-0.03	0.02	[-0.07, 0.01]	-0.001	0.02	[-0.04, 0.04]
Precentral Gyrus	-0.003	0.02	[-0.05, 0.03]	-0.04	0.02	[-0.09, -0.001]
Paracentral Lobule	-0.004	0.02	[-0.06, 0.03]	0.01	0.02	[-0.03, 0.06]
Frontal Pole	-0.004	0.03	[-0.07, 0.06]]	0.04	0.03	[-0.03, 0.1]
Superior Parietal Cortex	0.005	0.02	[-0.02, 0.03]	0.01	0.02	[-0.02, 0.04]
Inferior Parietal Cortex	0.005	0.02	[-0.02, 0.04]	0.03	0.02	[-0.01, 0.06]
Supramarginal Gyrus	-0.006	0.02	[-0.04, 0.03]	-0.004	0.02	[-0.04, 0.03]
Postcentral Gyrus	-0.004	0.02	[-0.03, 0.04]	-0.01	0.02	[-0.05, 0.03]

Precuneus Cortex	0.004	0.01	[-0.03, 0.02]	0.01	0.02	[-0.02, 0.04]
Superior Temporal Gyrus	-0.02	0.02	[-0.06, 0.02]	-0.03	0.02	[-0.08, 0.01]
Middle Temporal Gyrus	-0.004	0.02	[-0.04, 0.04]	-0.003	0.02	[-0.04, 0.04]
Inferior Temporal Gyrus	-0.004	0.02	[-0.03, 0.04]	-0.003	0.02	[-0.04, 0.04]
Banks of the Superior Temporal Sulcus	-0.02	0.02	[-0.07, 0.02]	-0.001	0.02	[-0.04, 0.04]
Fusiform Gyrus	-0.02	0.02	[-0.06, 0.01]	-0.004	0.02	[-0.04, 0.03]
Transverse Temporal Cortex	-0.04	0.03	[-0.1, 0.02]	-0.04	0.03	[-0.11, 0.01]
Entorhinal Cortex	-0.14	0.04	[-0.22, -0.05]	-0.05	0.04	[-0.14, 0.03]
Temporal Pole	-0.07	0.05	[-0.16, 0.03]	-0.04	0.05	[-0.14, 0.06]
Parahippocampal Gyrus	-0.09	0.03	[-0.16, -0.03]	-0.1	0.03	[-0.16, -0.04]
Lateral Occipital Cortex	-0.01	0.02	[-0.04, 0.02]	-0.01	0.02	[-0.05, 0.03]
Lingual Gyrus	-0.03	0.02	[-0.06, 0.01]	-0.004	0.02	[-0.04, 0.03]
Cuneus Cortex	-0.02	0.02	[-0.06, 0.02]	-0.02	0.02	[-0.07, 0.02]
Pericalcarine Cortex	-0.05	0.02	[-0.1, -0.01]	-0.03	0.03	[-0.08, 0.02]
Rostral Anterior Cingulate	-0.01	0.02	[-0.05, 0.04]	-0.04	0.03	[-0.1, 0.02]
Caudal Anterior-Cingulate Cortex	-0.05	0.03	[-0.11, 0.01]	-0.003	0.03	[-0.06, 0.05]
Posterior Cingulate Cortex	-0.04	0.02	[-0.08, 0.01]	-0.01	0.02	[-0.05, 0.03]
Isthmus Cingulate Cortex	0.01	0.02	[-0.03, 0.05]	-0.01	0.02	[-0.05, 0.03]
Insula Sulcus	-0.04	0.02	[-0.09, 0.01]	-0.04	0.03	[-0.09, 0.02]
Mean Thickness	-0.01	0.01	[-0.04, 0.01]	-0.01	0.01	[-0.03, 0.02]

Supplementary Table 3. Estimates and 95% CI for all FreeSurfer cortical thickness regions.

Region of Interest	Left			Right		
	Estimate	SD	95% CI	Estimate	SD	95% CI
Superior Frontal Gyrus	-1003	305	[-1569, -388]	-791	311	[-1373, -161]
Rostral Middle Frontal Gyrus	-209	245	[-712, 246]	-237	264	[-788, 270]
Caudal Middle Frontal Gyrus	-267	123	[-512, -20]	-118	148	[-414, 168]
Pars Opercularis	-327	106	[-538, -110]	-211	91	[-395, -43]
Pars Triangularis	-207	78	[-362, -57]	-181	102	[-372, 11]
Pars Orbitalis	-43	46	[-133, 40]	-91	57	[-200, 26]
Lateral Orbital Frontal Cortex	-253	111	[-468, -37]	-228	120	[-461, 10]
Medial Orbital Frontal Cortex	-195	88	[-365, -24]	-216	80	[-369, -55]
Precentral Gyrus	-438	230	[-882, -0.2]	-681	205	[-1085, -282]
Paracentral Lobule	-85	71	[-225, 57]	-67	89	[-234, 112]
Frontal Pole	-16	20	[-54, 21]	4	25	[-47, 53]
Superior Parietal Cortex	-118	243	[-588, 364]	84	244	[-411, 560]
Inferior Parietal Cortex	-192	265	[-708, 335]	-134	272	[-647, 422]
Supramarginal Gyrus	-88	251	[-595, 383]	73	190	[-281, 458]
Postcentral Gyrus	-169	172	[-504, 149]	-225	188	[-598, 150]

Precuneus Cortex	-253	165	[-565, 58]	-319	207	[-730, 71]
Superior Temporal Gyrus	-467	230	[-925, -37]	-530	216	[-954, -94]
Middle Temporal Gyrus	-294	228	[-769, 151]	-79	207	[-459, 334]
Inferior Temporal Gyrus	-243	210	[-669, 161]	-326	231	[-777, 112]
Banks of the Superior Temporal Sulcus	45	71	[-95, 181]	-4	56	[-117, 99]
Fusiform Gyrus	-105	156	[-432, 194]	-60	150	[-346, 240]
Transverse Temporal Cortex	-30	36	[-104, 38]	-43	23	[-89, -0.38]
Entorhinal Cortex	-172	50	[-262, -73]	-145	54	[-256, -42]
Temporal Pole	-100	64	[-224, 22]	-146	56	[-253, -31]
Parahippocampal Gyrus	-115	41	[-200, -38]	-79	39	[-157, -5]
Lateral Occipital Cortex	-230	209	[-638, 195]	-294	249	[-783, 209]
Lingual Gyrus	-122	144	[-400, 166]	-38	177	[-380, 310]
Cuneus Cortex	-82	86	[-258, 87]	-103	81	[-257, 60]
Pericalcarine Cortex	-161	92	[-358, 26]	-149	87	[-320, 19]
Rostral Anterior Cingulate	-109	71	[-244, 26]	-93	64	[-215, 24]
Caudal Anterior-Cingulate Cortex	-91	79	[-254, 58]	-33	75	[-182, 102]
Posterior Cingulate Cortex	-73	63	[-193, 54]	-66	69	[-197, 66]
Isthmus Cingulate Cortex	-2	52	[-96, 97]	-167	70	[-305, -39]
Insula Sulcus	-233	94	[-410, -49]	-405	119	[-635, -183]

Supplementary Table 4. Estimates and 95% CI for all FreeSurfer volume regions.
